# Revisiting enigmatic cortical calretinin-expressing interneurons

**DOI:** 10.3389/fnana.2014.00052

**Published:** 2014-06-24

**Authors:** Bruno Cauli, Xiaojuan Zhou, Ludovic Tricoire, Xavier Toussay, Jochen F. Staiger

**Affiliations:** ^1^Sorbonne Universités, UPMC University Paris 06, UM CR18, Neuroscience Paris SeineParis, France; ^2^Centre National de la Recherche Scientifique, UMR 8246, Neuroscience Paris SeineParis, France; ^3^Institut National de la Santé et de la Recherche Médicale, UMR-S 1130, Neuroscience Paris SeineParis, France; ^4^Institute for Neuroanatomy, UMG, Georg-August-University GöttingenGöttingen, Germany

**Keywords:** neuropeptides, neocortex, neocortical circuits, embryonic and fetal development, neuroenergetics

## Abstract

Cortical calretinin (CR)-expressing interneurons represent a heterogeneous subpopulation of about 10–30% of GABAergic interneurons, which altogether total ca. 12–20% of all cortical neurons. In the rodent neocortex, CR cells display different somatodendritic morphologies ranging from bipolar to multipolar but the bipolar cells and their variations dominate. They are also diverse at the molecular level as they were shown to express numerous neuropeptides in different combinations including vasoactive intestinal polypeptide (VIP), cholecystokinin (CCK), neurokinin B (NKB) corticotrophin releasing factor (CRF), enkephalin (Enk) but also neuropeptide Y (NPY) and somatostatin (SOM) to a lesser extent. CR-expressing interneurons exhibit different firing behaviors such as adapting, bursting or irregular. They mainly originate from the caudal ganglionic eminence (CGE) but a subpopulation also derives from the dorsal part of the medial ganglionic eminence (MGE). Cortical GABAergic CR-expressing interneurons can be divided in two main populations: VIP-bipolar interneurons deriving from the CGE and SOM-Martinotti-like interneurons originating in the dorsal MGE. Although bipolar cells account for the majority of CR-expressing interneurons, the roles they play in cortical neuronal circuits and in the more general metabolic physiology of the brain remained elusive and enigmatic. The aim of this review is, firstly, to provide a comprehensive view of the morphological, molecular and electrophysiological features defining this cell type. We will, secondly, also summarize what is known about their place in the cortical circuit, their modulation by subcortical afferents and the functional roles they might play in neuronal processing and energy metabolism.

## Introduction: what are the cortical calretinin-expressing interneurons?

Cortical calretinin (CR) expressing interneurons represent a heterogeneous subpopulation of about 10–30% of GABAergic interneurons (Kubota et al., 1994; Gonchar and Burkhalter, 1997; Tamamaki et al., 2003). They display different somatodendritic morphologies ranging from bipolar to multipolar (Jacobowitz and Winsky, 1991; Kubota et al., 1994; Gonchar and Burkhalter, 1997; Gonchar et al., 2008; Caputi et al., 2009). They are also diverse at the molecular level as they express numerous neuropeptides in different combinations including vasoactive intestinal polypeptide (VIP) (Kubota et al., 1994; Cauli et al., 1997), cholecystokinin (CCK) (Cauli et al., 1997; Gonchar et al., 2008), neurokinin B (NKB) (Kaneko et al., 1998; Gallopin et al., 2006) corticotrophin releasing factor (CRF) (Gallopin et al., 2006; Kubota et al., 2011), enkephalin (Enk) (Taki et al., 2000; Férézou et al., 2007), but also to a lesser extent and in a species-dependent manner neuropeptide Y (NPY) (Cauli et al., 1997; Wang et al., 2004; Gonchar et al., 2008) and somatostatin (SOM) (Cauli et al., 1997; Wang et al., 2004; Xu et al., 2010b). CR-expressing interneurons also exhibit different firing behaviors such as adapting, bursting or irregular (Kawaguchi and Kubota, 1996; Cauli et al., 1997, 2000; Porter et al., 1998; Wang et al., 2002; Karagiannis et al., 2009). They mainly originate from the caudal ganglionic eminence (CGE) (Xu et al., 2004; Butt et al., 2005) but a subpopulation also derives from the dorsal part of the medial ganglionic eminence (MGE) (Fogarty et al., 2007; Xu et al., 2008). Cortical GABAergic CR+ interneurons can be divided in two main populations: VIP-bipolar interneurons deriving from the CGE and SOM-Martinotti-like interneurons originating in the dorsal MGE. In the rodent neocortex, bipolar cells account for the majority of CR+ interneurons, therefore, we consider them as our main focus of the review. Due to the relatively few and scattered studies of CR+ interneurons, their inputs and outputs, the roles they play in cortical neuronal circuits as well as neuroenergetics of the brain remained elusive and enigmatic until quite recently. The aim of this review is, firstly, to provide a comprehensive view of the morphological, molecular and electrophysiological features defining cortical bipolar CR-expressing interneurons. We will, secondly, also review their places in the cortical circuit, their modulations by subcortical afferents and the functional roles they might play in neuronal processing and energy metabolism. We are convinced that the synthesis of recent data on bipolar CR-expressing interneurons will show that they are much less enigmatic than they appeared roughly 25 years ago (Peters and Harriman, 1988).

## Embryonic origins

**Figure 1:**
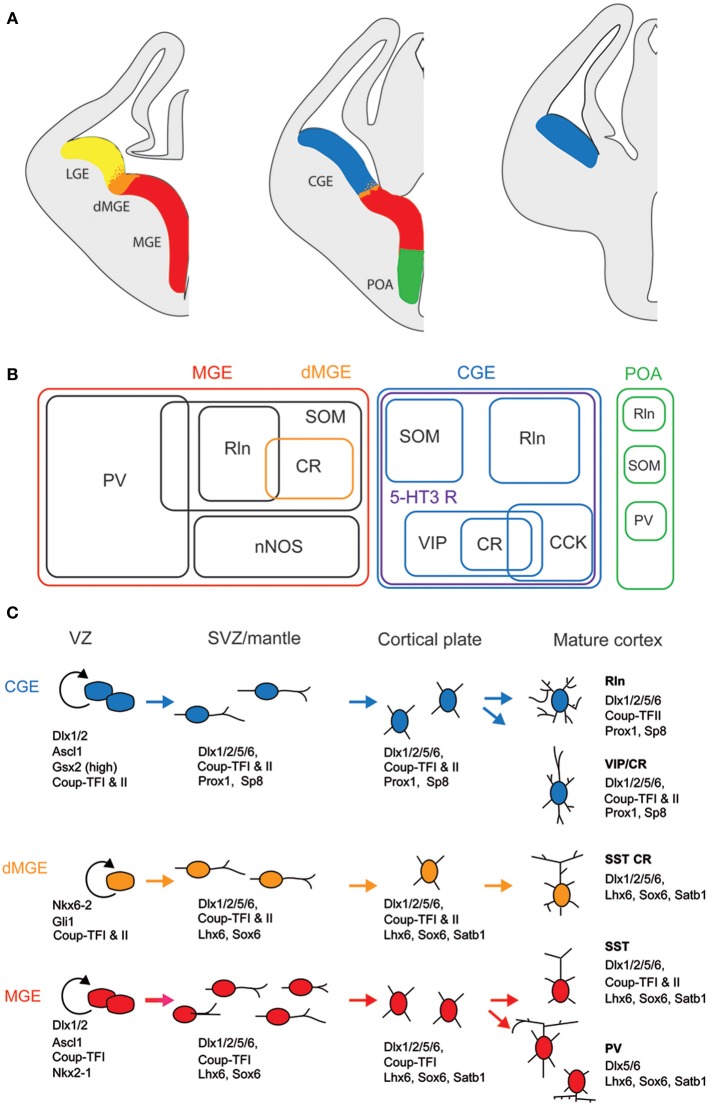
**Embryonic origins and genetic factors affecting the mature fate of cortical interneurons. (A)** Diagram showing the subdivisions of the embryonic telencephalon. The three regions where cortical and hippocampal interneurons originate are the medial ganglionic eminence (MGE) (including the dorsal MGE-dMGE), the caudal ganglionic eminence (CGE), and the preoptic area (POA). The lateral ganglionic eminence (LGE) gives rise, amongst other, to basal forebrain neurons. **(B)** Ball scheme of the major classes of cortical and hippocampal interneurons identified using neurochemical markers and represented depending of their place of origin in the embryonic telencephalon. The MGE generates 50% of all cortical interneurons and includes mainly parvalbumin (PV)-expressing and somatostatin (SOM)-expressing subtypes. In hippocampus, it also includes a large population expressing the neuronal isoform of nitric oxide synthase (nNOS). CGE-derived reelin (Rln)-expressing interneurons represent the neurogliaform cells. In both cortex and hippocampus, almost all CGE-derived interneurons express the type 3 serotonin receptor (5-HT3 R). In contrast with cortex, hippocampal SOM+ interneuron have a dual origin with a significant subset co-expressing 5-HT3 R. VIP; vasoactive intestinal polypeptide, CR, calretinin; CCK, cholecystokinin. **(C)** Genetic programs controlling neurogenesis, cell commitment, tangential, and radial migration and maturation of cortical interneuron. The subdivision of the neuroepithelium can be identified by combinatorial expression of transcription factors involved at different stages of cortical interneuron development. Some of these factors participate broadly in interneuron development such as Dlx and CoupTF gene families. Some transcription factors are unique to specific domains and/or stages of differentiation: Nkx2-1 defines the MGE and activates a cascade of genes including Lhx6, Sox6, and Satb1; Nkx6-2 and GLI1 are enriched in the dMGE. Prox1 and SP8 are expressed in CGE-derived cortical interneurons at all stages of their development (adapted from Kessaris et al., 2014).

Unlike glutamatergic neurons which are born in the ventricular zone of the telencephalic vesicle and then migrate radially (Molyneaux et al., 2007; Rakic, 2007), most of the GABAergic interneurons derive from one proliferative region called ganglionic eminence (GE) located in the ventral part of the telencephalon (Wonders and Anderson, 2006; Batista-Brito and Fishell, 2009; Bartolini et al., 2013). Once born, interneuron precursors migrate first dorsally, guided by attracting and repulsive molecular cues, then tangentially toward neocortex and hippocampus along two main migratory streams (Chedotal and Rijli, 2009; Marin, 2013). They eventually acquire their final laminar location by penetrating the cortical plate. Anatomically, the GE is subdivided in three main regions, the medial, lateral, and caudal GE (MGE, LGE, and CGE, Figure 1). Cortical and hippocampal interneurons derive mainly from the MGE and CGE, while the LGE is the major contributor of GABAergic interneurons of striatum and basal forebrain structures. The preoptic area (Figure 1) has recently been described as another source of cortical interneurons (Gelman et al., 2009) but given that very few CR+ interneurons derive from this zone, we will focus on the MGE and CGE.

Lineage analyses using either grafts or genetic tools such as Cre driver lines that label specifically a subfield of the GE have shown that parvalbumin-expressing (PV+) and SOM+ interneurons are generated in the MGE at different time points (between E9 and E15, Figure 1; Xu et al., 2004; Butt et al., 2005; Miyoshi et al., 2007; Tricoire et al., 2011). In contrast, similar approaches revealed that a large portion of the remaining interneuron subtypes, including CR+ interneurons, derive from the CGE and are produced at later embryonic stages (between E12.5 and E16.5, Figure 1; Lee et al., 2010; Miyoshi et al., 2010; Tricoire et al., 2011). In addition to being generated by distinct progenitors, CR+ interneuron precursors use a different migratory pathway. While SOM+ interneurons take a more rostrolateral route, CGE-derived interneurons take a caudal path for their tangential migration (Kanatani et al., 2008). The late birth of CGE-derived interneurons has the consequence that they are still migrating at birth and reach their final destination later than MGE-derived interneurons (Miyoshi et al., 2010). However, several refinements have to be considered. SOM+ hippocampal neurons exhibit a dual origin with one expressing type 3 serotonin receptor (5-HT3 R) and the other not (Chittajallu et al., 2013). In the neocortex, such a dichotomy has not been reported yet.

Several transcription factors of CR+ (and SOM+) interneurons are necessary for the proper specification and migration from the GE. Mice lacking the Dlx1 gene show reduction of CR+ and SOM+ interneurons without affecting the PV+ population (Cobos et al., 2005). Removal of the transcription factor Nkx2.1 restricted at early embryonic stages to the MGE domain (Figure 1) results in a molecular and cellular switch of MGE-derived cortical interneurons (PV+ and SOM+ subpopulations) to CGE-derived neurons (VIP+ and CR+ cells; Butt et al., 2008).

In contrast with SOM+ interneurons, little is known about the regional and cell type specification of CGE in general and of CR+ bipolar interneurons in particular. Gsx2 (also called Gsh2) is enriched in (but not restricted to) the LGE and CGE from early development (Figure 1) and has been directly implicated in promoting the CR+ interneuron identity (Xu et al., 2010a). The orphan nuclear receptor COUP-TFII shows restricted expression in the CGE (Kanatani et al., 2008; Willi-Monnerat et al., 2008) and, together with COUP-TFI, is required for the caudal migration of cortical interneurons (Tripodi et al., 2004). Moreover, in Nkx2.1 mutant mice, a higher number of CR+ and VIP+ cortical interneurons are generated and COUP-TFII is ectopically expressed in the MGE (Butt et al., 2008). Conversely, conditional loss-of-function of COUP-TFII in subventricular precursors and postmitotic cells leads to a decrease in VIP+ and CR+ interneurons, compensated by the concurrent increase of MGE-derived PV+ interneurons. Interestingly, COUP-TFI mutants are more resistant to pharmacologically induced seizures (Lodato et al., 2011). In addition to these genetic factors, electrical activity has been also shown to regulate development of cortical neurons. CR+ but not VIP+ interneurons activity is required before postnatal day 3 for correct migration and Elmo1, a target of Dlx1 and expressed in CR+ neurons, is both necessary and sufficient for this activity-dependent interneuron migration (De Marco Garcia et al., 2011).

Over the past decade, many advances have been achieved in the identification of the genetic factors that influence the specification of cortical and hippocampal interneurons especially for MGE-derived interneurons. However, the program specifying the identity of CGE-derived interneurons still needs to be unraveled. For instance, the cues that regulate the final maturation of the morphological, synaptic and electrophysiological properties have to be determined. Indeed, although embryonic origin is a major contributing factor, immature interneurons arriving at their final destination are likely to encounter local factors such as guidance molecules and specific levels of network activity that will instruct them where to grow dendrites and axons.

## Anatomical properties

### Laminar, columnar, and areal distribution pattern

**Figure 2:**
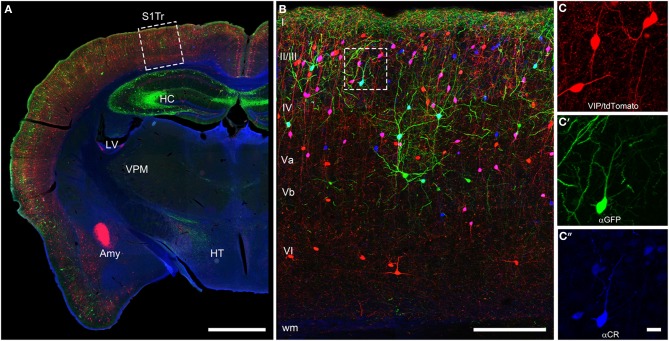
**Coronal brain section of a VIPcre/tdTomato/GIN mouse additionally stained for calretinin**. Immunostaining of calretinin in a coronal brain section of a VIPcre/tdTomato/GIN mouse shows VIP neurons in red, Martinotti cells in green and calretinin in blue in the primary somatosensory cortex (S1Tr). **(A)** Low magnification of one hemisphere depicting the hippocampus (HC), the lateral ventricle (LV), the amygdala (Amy), the thalamic nucleus ventralis posteromedialis (VPM), and the hypothalamus (HT); the *dashed rectangle* marks the selected area of **(B)**; scale bar: 1000 μm. **(B)** Close-up of the rectangle in **(A)** in a maximum intensity projection; *Roman numerals* indicate cortical layers; *dashed rectangle* marks the selected area of **(C–C″)**; scale bar: 250 μm. Please note that VIP neurons co-localizing CR appear pink whereas Martinotti cells that co-localize CR show a cyan-colored soma. **(C)** Red channel of the inset in **(B)**, showing the tdTomato signal only; **(C′)** green channel of the inset in **(B)**, showing the GFP only; **(C″)** blue channel of the inset in **(B)**, showing the labeled calretinin antibody only; scale bar for **(C–C″)** 20 μm. Please not that nearly all VIP and the single Martinotti cell are co-localizing CR.

The initial descriptions of the brain-wide distribution of CR+ neurons did not report obvious differences in the number or type of neurons across the different cortical areas (Jacobowitz and Winsky, 1991; Resibois and Rogers, 1992; Gabbott et al., 1997). They observed that within a certain cortical area always supragranular layers II/III show the highest density of CR+ neurons (Figure 2). A more fine-grained analysis, however, suggested that there is a subtle gradient from rostral to caudal, with a higher density of CR+ neurons in the visual cortex (Xu et al., 2010b), a finding similar to that of VIP+ neurons, one of the major subtypes of CR+ neurons (Morrison et al., 1984; Rogers, 1992; Gonchar et al., 2008). Interestingly, in the mouse barrel cortex where columnar modules are easily visualized, both, CR+ and VIP+ interneurons showed a preference for septal compartments over barrel-associated columns (Zilles et al., 1993; Melvin and Dyck, 2003). Such a preference was not obvious for the rat barrel cortex (Bayraktar et al., 2000), although no tangential sections where used, which would have allowed a much better resolution of columnar compartments. In summary, CR+ neurons show a relatively uniform appearance across cortical areas in terms of distribution, numbers and cell types, suggesting that they perform a basic and comparable function in neuronal processing or control of energy supply.

### Cellular morphology

Since the recognition of cortical interneurons as a distinct cell class different from principal (i.e., pyramidal) cells (Jones, 1975; Fairen et al., 1984; Ramón y Cajal, 1995), researchers have been struck by the abundance of morphological features that are already expressed at the somatodendritic level, let alone by the manifold axonal ramification patterns when they later became observable (DeFelipe et al., 2013). To agree upon a common nomenclature on the somatodendritic patterns of GABAergic interneurons, a minimal consensus paper was published (Ascoli et al., 2008). However, we feel that for the appreciation of the full diversity of observable somatodendritic configurations, this terminology has to be extended and refined in the future. In the following, we will elaborate on what we originally proposed for VIP+ neurons in the rat barrel cortex (Bayraktar et al., 2000).

**Figure 3:**
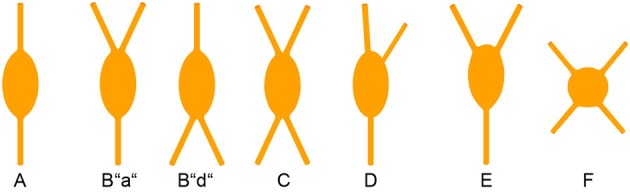
**Somatodendritic morphology of CR+ interneurons**. For details see text.

This classification is strictly focused on the *origin* of the primary dendrites at the soma (Figure 3). Specific examples in a histological preparation can be found in Figure 2.

- **Bipolar:** Two dendrites originating from opposite sides of a spindle (to round)-shaped soma. It is not important in this classification how close or far from the soma these dendrites starts to branch into their terminal tufts.
- **Single tufted:** A single dendrite on one side of the soma and *at least 2* dendrites originating individually from the opposite side. We suggest that acceptable origins from where a dendrite is allowed to emerge for the tufted category are ±20° from the upper or lower pole of the soma. (In case that there are dendritic origins outside these sectors, the neuron belongs to one of the following groups). This group can be further specified as “a” = tuft ascending and “d” = tuft descending.
- **Bitufted:** Two dendritic tufts as defined above, originating from opposite sides of the soma.
- **Modified:** Bipolar/single-tufted/bitufted: they possess a third dendrite originating anywhere else than the above defined circumference at the soma.
- **Tripolar:** This is a difficult term since as such it can be a modified bipolar with a more or less extensive third dendrite (that should also be vertically oriented) or it can be the “smallest” form of a multipolar cell (which then can be oriented to any direction). Whenever this term is used, it should be specified to which of the more general categories of somatodendritic configuration (D or F) the cell is closer.
- **Multipolar:** At least 4 (or 3, see above) dendrites originating from a round to polygonal soma.
- **Others:** Any neuron that is so different and rare (i.e., horizontal) that it does not fit in the above categories.

With this in mind, we here shortly summarize that cortical CR+ neurons, on the basis of their somatodendritic properties, were classified by different researchers as bipolar (Jacobowitz and Winsky, 1991; Kawaguchi and Kubota, 1996), bipolar and multipolar (Resibois and Rogers, 1992), or bipolar, bitufted, multipolar and horizontal (Caputi et al., 2009; Barinka and Druga, 2010), respectively. Unfortunately, not too many axonal reconstructions of these cells are available but it is assumed that the bipolar or bitufted dendritic trees are mostly accompanied by a vertical translaminar axonal arbor whereas the multipolar dendritic trees go along with a horizontal transcolumnar ramification of the axon (Caputi et al., 2009).

## Neurochemical properties: co-expression of neuropeptides and classical neurotransmitters

The degree of co-expression of CR with other markers can strongly vary between species. For instance in rat, a large majority (70–90%) of cortical CR+ cells exhibits VIP immunoreactivity (Rogers, 1992; Kubota et al., 1994) whereas this co-expression drops down to about 35% in mouse (Gonchar et al., 2008; Xu et al., 2010b). This difference probably comes from the presence of a SOM+ neuronal subpopulation accounting for 30–40% of CR+ cells in mouse (Halabisky et al., 2006; Xu et al., 2006; Gonchar et al., 2008), which is virtually absent in rat (Rogers, 1992; Kubota et al., 1994, 2011; Gonchar and Burkhalter, 1997) and human (Gonzalez-Albo et al., 2001). However, such a difference between species is not retrieved at the mRNA level since co-expression of CR and SOM transcripts has been observed using single cell RT-PCR both in mouse (Perrenoud et al., 2013) and rat in which the co-expression level is up to 40% of the CR+ cortical neurons (Cauli et al., 1997, 2000; Wang et al., 2004; Toledo-Rodriguez et al., 2005; Gallopin et al., 2006; Pohlkamp et al., 2013). The absence of co-immunodetection of CR and SOM in rat is probably due to a species-dependent post-transcriptional (Kwan et al., 2012) and/or a post-translational control (Herrero-Mendez et al., 2009). These observations indicate that CR-expressing bipolar cells, but not CR+/SOM+ (often multipolar) cells, are a common cell type in rats and mice.

**Figure 4:**
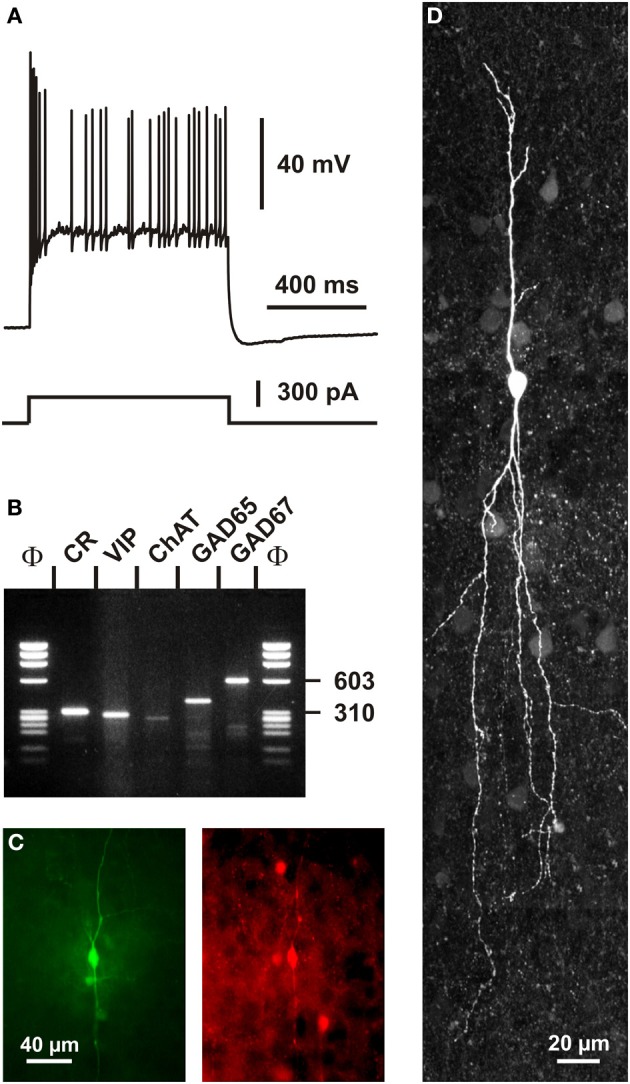
**Single cell RT-PCR analysis of a rat CR+ interneuron. (A)** Current-clamp recording obtained in response to a depolarizing current pulse (300 pA). Note the initial burst followed by irregularly discharged action potentials. **(B)** Single-cell RT-PCR analysis of the same neuron revealing co-expression of CR, VIP, ChAT, GAD65, and GAD67. **(C)** Comparison of the biocytin labeling of the recorded neuron (left panel) with the immunostaining of the slice with an antibody against CR (right panel). Note the immunoreactivity of the biocytin-labeled cell (scale bar 40 μm, adapted from Cauli et al., 1997). **(D)** Intracellular biocytin labeling of another CR+ bipolar cell analyzed by singe cell RT-PCR. This neuron had a vertically oriented dendritic arborization. Pial surface is upward (scale bar, 20 μm).

In addition to VIP (Rogers, 1992; Kubota et al., 1994; Halabisky et al., 2006; Xu et al., 2006; Gonchar et al., 2008), CR+ bipolar interneurons co-express other neuropeptides (Figure 1). CRF and the preprotachykinin Neurokinin B (NKB), expressed in about one third of CR+ bipolar neurons, are rather common neuropeptides in superficial layers (Kaneko et al., 1998; Gallopin et al., 2006; Kubota et al., 2011). Enkephalin, an endogeneous opioid, is also expressed in a subpopulation of CR+/VIP+ bipolar interneurons (Taki et al., 2000; Férézou et al., 2007). By contrast, despite its abundance in the cerebral cortex (Beinfeld et al., 1981) and its frequent co-expression with VIP (Cauli et al., 1997, 2000; Kubota and Kawaguchi, 1997; Férézou et al., 2002; Kubota et al., 2011), CCK is only expressed in a minority of CR+ neurons (Cauli et al., 1997; Kubota and Kawaguchi, 1997; Gallopin et al., 2006; Gonchar et al., 2008; Karagiannis et al., 2009; Kubota et al., 2011; Pohlkamp et al., 2013). In addition to neuropeptides, up to 50% of VIP+ bipolar neurons co-express choline acetyl-transferase (ChAT) (Eckenstein and Baughman, 1984; Peters and Harriman, 1988; Chédotal et al., 1994a; Bayraktar et al., 1997; Cauli et al., 1997; Porter et al., 1998; Von Engelhardt et al., 2007; Gonchar et al., 2008; Consonni et al., 2009). Although co-expression of GABA and ChAT has been demonstrated in CR+/VIP+ bipolar interneurons (Kosaka et al., 1988; Bayraktar et al., 1997; Cauli et al., 1997; Porter et al., 1998) (Figure 4), the cholinergic nature of bipolar neurons is species-dependent. Indeed, the vesicular acetylcholine transporter, an essential component of the cholinergic system, is expressed in cortical interneurons from rats but not from mice or humans (Schafer et al., 1994, 1995; Gilmor et al., 1996; Weihe et al., 1996; Bhagwandin et al., 2006). Expression of nitric oxide synthase (NOS) is very marginal in bipolar CR+/VIP+ neurons and also appears to occur mainly in mouse (Lee and Jeon, 2005; Gonchar et al., 2008; Tricoire et al., 2010; Magno et al., 2012; Perrenoud et al., 2012a; Pohlkamp et al., 2013).

In summary, CR+/VIP+ bipolar neurons express a large repertoire of neurotransmitters and neuromodulators indicative of their neurochemical diversity. This suggests that they are likely to play multiple roles in cortical physiology.

## Electrophysiological features

**Figure 5:**
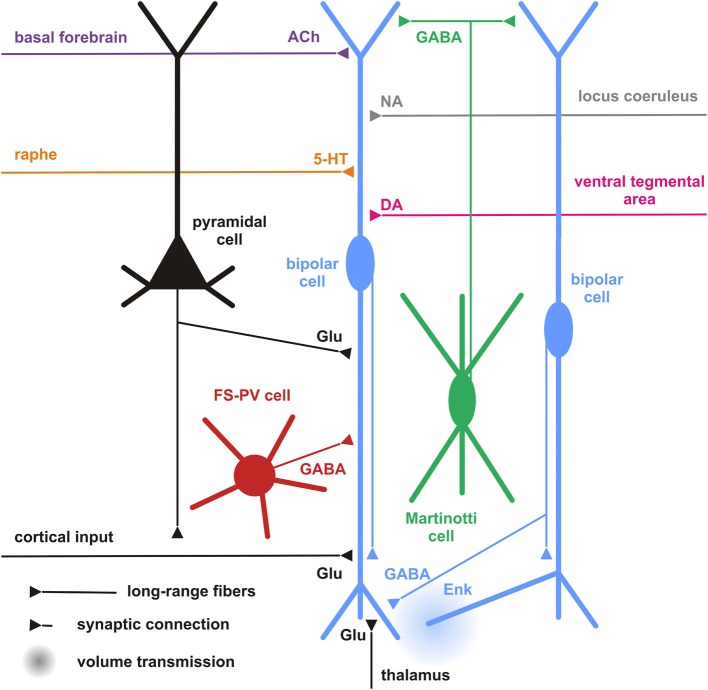
**Cortical and subcortical inputs of CR+ bipolar interneurons**. Schematic representation summarizing the different local cortical neurons (colored cells) and the long-range cortical and subcortical axon terminals (colored fibers) targeting CR+ bipolar interneurons (centered blue bipolar cell). Dendrites and axons are depicted by thick and thin lines, respectively. Anatomical synaptic connections are represented by triangles and putative volume transmission by spherical gradients. The local neuronal types targeting bipolar CR+ and their respective neurotransmitters are schematized and color-coded; pyramidal cells (black, glutamate), fast spiking (FS)-PV neurons (red, GABA), Martinotti cells (green, GABA) other CR+ bipolar interneurons (blue, GABA and Enk [enkephalin]). Neurotransmitters of the long-range cortical and subcortical fibers are color-coded (Glu [glutamate; Black], 5-HT [serotonin, orange], ACh [acetylcholine, purple], NA [noradrenaline, gray], DA [dopamine, pink]) and their major origins indicated using the same color code.

**Figure 6:**
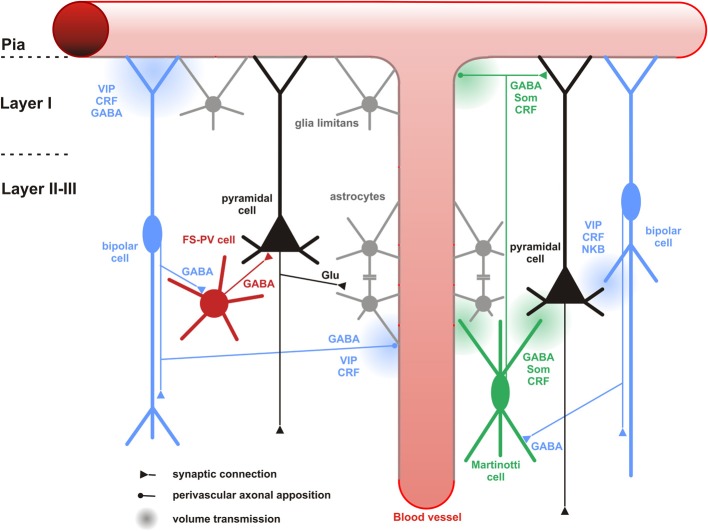
**Neuronal and non-neuronal targets of CR+ bipolar cells**. Schematic representation summarizing the different neuronal and non-neuronal targets of CR+ bipolar interneurons (blue neurons) and Martinotti cells (green neuron). Dendrites and axons are depicted by thick and thin lines, respectively. Anatomical synaptic connections are represented by triangles, axonal perivascular appositions by dots and putative volume transmission by spherical gradients. The cell types targeted by CR+ neurons are schematized and color-coded; FS-PV neurons (red), Martinotti cells (green), pyramidal cells (black), glial cells (gray). Astro-glial coverage of blood vessels is illustrated by a gray-colored abluminal side. The specific action of the multiple neurotransmitters released by CR+ neurons is depicted by their location near the specific target and their origin color-coded (bipolar cell, blue; Martinotti cells, green). VIP, vasoactive intestinal polypeptide; CRF, corticotrophin releasing factor; NKB, neurokinin B; Som, somatostatin. The disinhibitory action of bipolar cells is represented by disynaptic circuits involving a bipolar interneuron, an intermediary interneuron (left: FS-PV cell; right: Som-Martinotti cell) and a pyramidal cell, respectively.

A remarkable passive electrophysiological feature (Ascoli et al., 2008) of CR+/VIP+ bipolar neurons is their relatively high input resistance (Kawaguchi and Kubota, 1996; Cauli et al., 1997, 2000; Gallopin et al., 2006; Karagiannis et al., 2009; Lee et al., 2010; Vucurovic et al., 2010), contrasting sharply with the low input resistance of fast spiking PV+ neurons (Kawaguchi and Kubota, 1993; Okaty et al., 2009; Battaglia et al., 2013). This property allows CR+/VIP+ neurons to be substantially depolarized by the small excitatory synaptic currents they receive from thalamic inputs (Lee et al., 2010). The presence of a prominent I_*H*_ current in SOM+ neurons underlying their distinctive voltage sag induced by hyperpolarization partially explains the slightly lower input resistance and the more depolarized resting membrane potential of SOM+ interneurons compared with those of CR+/VIP+ bipolar neurons (Wang et al., 2004; Halabisky et al., 2006; Ma et al., 2006; Xu et al., 2006; Karagiannis et al., 2009). Alike SOM+ interneurons, VIP+ cells have been reported to exhibit the ability to discharge low-threshold spikes driven by I_*T*_ calcium channels (Kawaguchi and Kubota, 1996; Cauli et al., 1997; Porter et al., 1999; Wang et al., 2004). VIP+ bipolar interneurons displayed action potentials of a duration intermediate to those of fast spiking interneurons and pyramidal cells (Kawaguchi and Kubota, 1996; Cauli et al., 1997, 2000; Karagiannis et al., 2009). A complex repolarization phase of their action potentials consisting of a fast after-hyperpolarization (AHP), followed by an after-depolarization and a medium AHP has been frequently observed in both VIP+ and SOM+ interneurons (Wang et al., 2004; Fanselow et al., 2008; Karagiannis et al., 2009), VIP+, but also SOM+ interneurons, characteristically displayed a pronounced frequency adaptation (Kawaguchi and Kubota, 1996; Cauli et al., 1997, 2000; Wang et al., 2004; Halabisky et al., 2006; Ma et al., 2006; Xu et al., 2006) which can result in an irregular firing pattern (Figure 4) (Cauli et al., 1997, 2000; Wang et al., 2004) when a slowly inactivating I_*D*_ potassium current is prominently present (Porter et al., 1998). Similarly to SOM+ interneurons, CR+ bipolar neurons exhibit backpropagating action potentials accompanied by an intracellular Ca^2+^ increase (Kaiser et al., 2001; Goldberg et al., 2003; Cho et al., 2010), which allows the release of neurotransmitters from dendrites as reported for SOM+ interneurons (Zilberter et al., 1999). Since the extent of action potential backpropagation is modulated by the presence of I_*A*_ potassium channels (Goldberg et al., 2003; Cho et al., 2010), this suggests that the dendritic release of CR+ bipolar interneurons (Figures 5, 6) can be finely tuned.

In summary, CR+ bipolar interneurons largely display the electrophysiological features of adapting neurons (Ascoli et al., 2008). Their distinctive intrinsic electrophysiological properties indicate that they have specific integrative properties and therefore are likely to play specific roles in the physiology of cortical circuits, e.g., state-dependently remove the blanket of inhibition from principal neurons (Karnani et al., 2014).

## Synaptic inputs

### Cortical

In general, our specific knowledge on connectivity is very restricted. Probably the most precise data for local synaptic inputs to CR+ neurons come from paired recordings in layer II/III of the primary somatosensory cortex in a CR-BAC-transgenic mouse (Caputi et al., 2009). These authors reported that the two types of neurons that they defined as the major cellular components of the CR+ neuronal population receive different types of inputs (and also form different types of output; see below). Bipolar VIP+/CR+ interneurons (BCR) receive functionally depressing inputs from local pyramidal cells with a probability of 18.3%, from fast-spiking interneurons (29.7%), from multipolar SOM+/CR+ interneurons (MCR; 41.1%) as well as facilitating inputs from other BCR (31.8%) (Figure 5). By contrast, MCR show facilitating inputs from pyramidal cells (17.4%) and from BCR (76.4%) as well as depressing inputs from fast-spiking cells (20%) and other MCR (9.8%). Interestingly, it appears that inputs to CR+/VIP+ (BCR) neurons are mainly depressing (Porter et al., 1998; Rozov et al., 2001), whereas all their outputs are facilitating and, vice versa, for CR+/SOM+ (MCR) neurons inputs are often facilitating whereas their outputs show a target cell type-specific variability (Caputi et al., 2009). These data do not agree well with paired recordings from rat barrel cortex, probably due to a mixture of species differences and less defined populations of bipolar vs. multipolar neurons in this study (Reyes et al., 1998).

Another rich source of observations describing the inputs to layer II/III CR+ neurons is provided by the glutamate uncaging studies in different transgenic mouse lines (Xu and Callaway, 2009). They distinguished CR+ and CR- Martinotti cells and (putatively CR+ and VIP+) bipolar cells. CR+ Martinotti cells (as all other types of inhibitory neurons in their study) received their strongest excitatory inputs within layer II/III whereas bipolar cells possessed a second strong origin of inputs located in layer IV.

Going beyond the local cortical circuits, Gonchar and Burkhalter also described long-range cortical inputs to CR+ neurons (Figure 5) (Gonchar and Burkhalter, 2003). Here, tracer injections to label connections between primary (V1) and secondary (V2) visual areas disclosed a circuit motif where CR+ neurons (specifically in layer I) are involved in feedback inhibition from V2 to V1, whereas few if any such long range connections are found on layer II/III CR interneurons. In this layer, PV+ interneurons were the main recipients for feedback projections, as they were for V1 to V2-directed feedforward projections.

CR+ bipolar interneurons may integrate these glutamatergic excitatory afferents by expressing ionotropic glutamate receptors of the AMPA subtype exhibiting a low Ca^2+^ permeability (Porter et al., 1998; Rozov et al., 2001) associated with high levels of the GluR2 subunit (Jonas et al., 1994). They, however, distinctly exhibit a low occurrence of GluR3 subunits and mainly express the flop variants of GluR subunits (Lambolez et al., 1996; Angulo et al., 1997; Porter et al., 1998; Cauli et al., 2000). GluR5 and GluR6 are the main kainate receptor subunits expressed in CR+ bipolar neurons (Porter et al., 1998; Cauli et al., 2000). Similarly to other cortical interneurons, CR+ bipolar interneurons express NR2A, B and D subunits of the NMDA receptor, the latter being found at very low levels in pyramidal cells (Flint et al., 1997; Porter et al., 1998; Cauli et al., 2000), indicating that bipolar interneurons can express very slowly inactivating NMDA receptors (Vicini et al., 1998). Similarly to SOM+ interneurons, the activity of CR+ bipolar and can be also modulated by the group I metabotropic glutamate receptors mGluR1 and mGluR5 (Baude et al., 1993; Kerner et al., 1997; Cauli et al., 2000; van Hooft et al., 2000; Ferraguti et al., 2004). The group III metabotropic receptor mGluR7 expressed by CR+/VIP+ bipolar neurons (Cauli et al., 2000) is presynaptically localized onto synapses targeting SOM+ interneurons which suggests a glutamatergic control of disinhibition (Dalezios et al., 2002). In summary, CR+/VIP+ bipolar neurons exhibit a cell-type-specific expression pattern of glutamate receptors allowing them to integrate glutamatergic inputs with distinct temporal and spatial features (Figure 5).

In terms of their inhibitory inputs, alike SOM+ cells, CR+ bipolar interneurons sampled from layers II/III, IV, and V, which was quite uncommon for most other cell types studied (Xu and Callaway, 2009). In their study, Gonchar and Burkhalter (1999) corroborated with morphological methods that rat CR+ neurons in layer II/III do innervate each other with numerous GABAergic (symmetric) synapses on the soma as well as proximal and distal dendrites. In addition, many symmetric and asymmetric synapses that were not further identified by their specific origin could be found on the CR+ neurons. This is very similar to what we have described for VIP+ interneurons in rat barrel cortex (Staiger et al., 2004).

Since the expression of GABA receptor subunits by specific cortical neuronal subtypes is still poorly documented (Ruano et al., 1997; Olah et al., 2009) and given the diversity of these subunits (Laurie et al., 1992; Pirker et al., 2000), the specific GABA-A (but also GABA-B) receptor subunits of CR+ bipolar interneurons remain largely undetermined.

Bipolar interneurons also possess receptors for intrinsic cortical neuromodulators including opioids and endocannabinoids. For instance, in the cerebral cortex μ-opoid receptors are mainly expressed by bipolar interneurons which also produce enkephalin, its endogenous agonist (Taki et al., 2000). This autocrine/paracrine μ-opoid transmission has been shown to restrict the inhibitory drive of CR+ bipolar interneurons onto pyramidal cells (Férézou et al., 2007) (Figure 5). Similarly, the endocannabinoid transmission has been shown to exert a long-lasting self-inhibition of putative SOM+ neurons (Bacci et al., 2004) by activation of CB1 receptors, which are broadly expressed by cortical neurons (Bodor et al., 2005; Hill et al., 2007). These two neuromodulatory systems, recruited during sustained firing, provide activity-dependent negative feedback to restrict the inhibitory actions of CR+ interneurons.

In summary, CR+ bipolar neurons possess a large integrative capability for excitatory as well as inhibitory inputs across different layers of a cortical column. A lot remains to be done to obtain a conclusive picture of the cortical input connectivity of CR+ bipolar neurons. However, already with the limited information available, it appears that these GABAergic interneurons do not only integrate excitatory and inhibitory inputs from local origin but that they also process long-range inputs from within the cortex and possibly subcortical sources (see next sections).

### Subcortical inputs

#### Serotonergic

Due to the paucity of specific studies on subcortical targeting of cortical bipolar CR+ interneurons, inputs to the partially overlapping populations of VIP+ as well as 5-HT3 R+ interneurons might be considered here, too (Paspalas and Papadopoulos, 2001; Férézou et al., 2002; Cauli et al., 2004; Lee et al., 2010; Vucurovic et al., 2010; Rudy et al., 2011). The median raphe nucleus was found to be the subcortical region that gives rise to serotonergic input to CR+ interneurons in rat hippocampus (Acsady et al., 1993). By using a bridge protein combined with retrograde viral tracers, the paramedian raphe was also found to project to a subpopulation of interneurons, which express ErbB4 in mouse primary somatosensory cortex (Choi and Callaway, 2011). Selectivity of the TVB-NRG1 bridge protein restricted the transfection to the subgroups of interneurons immunopositive for VIP and/or CR as well as ErbB4, including also small numbers of PV+/ErbB4 neurons but not SOM+ cells (Choi et al., 2010; Xu et al., 2010b).

Although the expression of the ionotropic 5-HT3 R is restricted to a subset of cortical interneurons, including CR+ bipolar neurons and neurogliaform cells (Morales and Bloom, 1997; Férézou et al., 2002; Lee et al., 2010; Vucurovic et al., 2010), a subpopulation of hippocampal SOM+ O-LM cells originating from the CGE was recently found to be 5-HT3 R+ (Chittajallu et al., 2013). Cell-type-specific expression patterns of metabotropic 5-HT receptors are by far less documented. Nevertheless, 5-HT1a and 5-HT2a receptors are largely co-expressed in pyramidal cells and in a minority of interneurons positive for PV+ or CB+ (Aznar et al., 2003; Santana et al., 2004), suggesting that CR+ bipolar interneurons barely express these receptors. Similarly, 5HT-2c R is not prominently expressed in CR+ neurons of the rat medial prefrontal cortex (Liu et al., 2007). Given the reported segregation of 5-HT3 and 5-HT2a receptors in the monkey cerebral cortex (Jakab and Goldman-Rakic, 2000), it appears that 5-HT3 R is the major serotonin receptor expressed by CR+ bipolar interneurons. These observations indicate that serotonergic raphe neurons can rapidly activate CR+ bipolar interneurons as shown in the cerebral cortex (Férézou et al., 2002) (Figure 5).

Besides the paramedian raphe, additional origins of input were detected (with a decreasing probability) in the thalamus, secondary somatosensory, ipsilateral motor, retrosplenial, and contralateral primary somatosensory cortex as well as the nucleus basalis of Meynert (Choi et al., 2010; Xu et al., 2010b). Amongst the detected brain regions, the thalamus and basal nucleus of Meynert have been mostly investigated.

#### Thalamic

By anterograde tracing with PHA-L, neurons in the thalamus (mainly ventral posterior nucleus and lateral geniculate nucleus) were approved to form synaptic terminals onto the cortical VIP+ population of inhibitory interneurons (Staiger et al., 1996; Hajos et al., 1997). These anatomical observations were corroborated by functional evidence showing that 5-HT3 R+ interneurons receive monosynaptic thalamocortical inputs (Lee et al., 2010). By contrast, putative SOM+ interneurons were found to barely receive direct thalamic inputs (Beierlein et al., 2000, 2003).

#### Cholinergic

The projections from the basal nucleus of Meynert, where most of the cholinergic neurons projecting to the neocortex are located, are probably correlated to the nicotinic responsiveness of VIP+/5-HT3 R+ bipolar interneurons (Porter et al., 1999; Férézou et al., 2002, 2007; Lee et al., 2010; Arroyo et al., 2012; Fu et al., 2014). CR+/VIP+ bipolar neurons express functional high-affinity nicotinic receptors (Porter et al., 1999; Férézou et al., 2002, 2007; Gulledge et al., 2006; Lee et al., 2010; Arroyo et al., 2012; Fu et al., 2014) composed of α4β 2 subunits and to a lesser extent α5 subunit, a composition which is developmentally regulated (Winzer-Serhan and Leslie, 2005). Alike SOM+ interneurons, VIP+ bipolar neurons are depolarized by muscarinic agonists (Kawaguchi, 1997; Fanselow et al., 2008). Expression of m2 receptors has been reported in a minority CR+ interneurons of the rat entorhinal cortex (Chaudhuri et al., 2005) but the subtype-specific identity of the muscarinic receptors inducing the depolarization of VIP+ bipolar interneurons remains unknown. This indicates that CR+ interneurons integrate cholinergic afferences from the basal forebrain (Figure 5) as recently shown for ChAT+ bipolar interneurons using optogenetics (Arroyo et al., 2012), which might be important for behavioral state-dependent control of cortical circuits (Pi et al., 2013).

#### Noradrenergic

About 20% of VIP+ interneurons are contacted by noradrenergic fibers (Figure 5), however, often with only a single symmetric synapse (Paspalas and Papadopoulos, 1998, 1999; Toussay et al., 2013). Despite the described expression of α- and β-adrenoreceptors in the cerebral cortex (Nicholas et al., 1993a,b; Pieribone et al., 1994; Scheinin et al., 1994; Venkatesan et al., 1996) and reported excitatory effects of α-adrenoreceptors in hippocampal and cortical interneurons (Bergles et al., 1996; Marek and Aghajanian, 1996; Kawaguchi and Shindou, 1998), the expression of adrenoreceptors in CR+ interneurons has not been specifically investigated. However, inhibitory effects of α-adrenoreceptors have been observed in discrete subpopulations of CCK+ and SOM+ interneurons (Kawaguchi and Shindou, 1998), whether or not this inhibitory effect of α-adrenoreceptors is existent in CR+ bipolar interneurons remains unknown. β 1- and β 2-adrenoreceptors are not as frequently expressed in CR+ interneurons as compared to other types of interneurons (Liu et al., 2014).

#### Dopaminergic

Dopaminergic terminals have been shown to target interneurons (Sesack et al., 1995b), specifically CR+ interneurons in monkey prefrontal cortex (Sesack et al., 1995a) (Figure 5). However, there is no clear segregation in the expression profiles of dopamine receptors, which are frequently observed in pyramidal cells and interneurons of rodent and primate cortex with substantial co-expression (Vincent et al., 1993; Khan et al., 1998, 2000; Ciliax et al., 2000; Wedzony et al., 2000; Rivera et al., 2008; Oda et al., 2010). Nevertheless, regarding the expression of D1-like receptors, D1 receptors predominate in PV+ interneurons of the primate prefrontal cortex whereas it is D5 in CR+ interneurons (Glausier et al., 2009).

At the moment, all that can be stated with certainty is that a lot of work is still needed to get a better idea how cortical CR+ bipolar interneurons are influenced by subcortical sensory or modulatory inputs (Figure 5), which receptors are involved and how this modulation specifically affects sensory information processing or more general brain-state activity.

## Targets and functions

### Cortical circuit

#### Neuronal targets

Hippocampal CR+ interneurons are considered to be interneuron-specific interneurons (Klausberger and Somogyi, 2008), which preferentially or even exclusively target other interneurons, presumably on their dendritic shafts. In rat hippocampus, post-embedding immunogold studies showed that most of the targets of CR+ and VIP+ boutons (which might be derived from the same bipolar neurons co-localizing CR and VIP) were GABAergic dendrites (Acsady et al., 1996a; Gulyas et al., 1996). In the neocortex, the GABAergic dendrite targeting property of CR+ or VIP+ interneurons is more complex when taking cortical areas and layers into account. In monkey and rat visual cortex, differential modes of innervation by CR+ interneurons exist in different layers. In layers I-III, CR+ interneurons tended to synapse on dendritic shafts of other not further specified GABAergic interneurons. However, in layers IV-VI, GABA-negative spines belonging to pyramidal neurons were primarily innervated (Meskenaite, 1997; Gonchar and Burkhalter, 1999).

Paired recordings in layer II/III of the primary somatosensory cortex of CR-BAC-transgenic mice (Caputi et al., 2009) gave an idea of the functional output of these CR neurons. Bipolar CR+ (BCR) innervate pyramidal cells (11.6%), fast-spiking interneurons (29.7%), other BCR (31.8%) and as already mentioned, preferably MCR (76.4%). It is remarkable that the input with the highest probability in this study represents indirect evidence for the recently disclosed disinhibitory circuit from VIP neurons to Martinotti cells (Figure 6), for which several direct (Lee et al., 2013; Pfeffer et al., 2013; Pi et al., 2013; Fu et al., 2014) and indirect (Staiger et al., 2004; Gentet et al., 2012) lines of evidence are now available.

It has been shown in both, hippocampus and somatosensory cortex of rodents, that calbindin-expressing (CB+) interneurons are the targets of CR+ or VIP+ interneurons (Acsady et al., 1996b; Gulyas et al., 1996; Staiger et al., 2004). CB+ interneurons form a heterogeneous population and can coexpress other neuropeptides or other calcium binding proteins, thus, it is reasonable to further differentiate CB+ interneurons in order to distinguish the CR+ or VIP+ outputs to specific sets of interneurons. The most likely contacts are made with Martinotti cells which coexpress the markers CB and SOM (Kawaguchi and Kubota, 1996; Wang et al., 2004). Supporting this possible connection, recent functional studies described that in mouse somatosensory, visual, auditory, and prefrontal cortex, SOM+ interneurons were preferentially innervated by local VIP+ interneurons (Lee et al., 2013; Pfeffer et al., 2013; Pi et al., 2013). Because of the diversity of VIP+ and SOM+ neuronal subgroups, it would be important to know what exact types in terms of morphology contribute to this microcircuit. Some indirect evidence already exists. Bipolar CR+ interneurons (BCRs) were immunopositive for VIP while multipolar CR+ interneurons (MCRs) were not (Caputi et al., 2009) and subgroups of SOM+ interneurons coexpressing CR with up to 95% or more than 50% were found in mouse visual and somatosensory cortex layers I–III, respectively (Xu et al., 2006). Therefore, it is hypothesized that BCRs, which are presumably CR+/VIP+ bipolar interneurons, target MCRs, which are probably CR+/SOM+ Martinotti interneurons.

Beside Martinotti interneurons, subgroups of basket cells such as large basket cells and nest basket cells could be immunopositive for CB, however, it is much more likely that these co-localize PV and not SOM (Wang et al., 2002). Two anatomical studies showed that VIP+ interneurons innervated PV+ interneurons, although it is debated whether there exists a preferential targeting in terms of putative PV+ subgroups (Dávid et al., 2007; Hioki et al., 2013). Behavioral experiments on mouse auditory and prefrontal cortex found that next to SOM+ interneurons, PV+ interneurons were the second major outputs of VIP+ interneurons (Pi et al., 2013) (Figure 6), lending further support to a functional role of the above mentioned anatomical connections. In summary, the CR+ bipolar interneurons seem to preferentially but not exclusively target other GABAergic interneurons, the molecular and anatomical properties of which need to be better analyzed.

#### Neuronal processing

The input-output connectivity pattern described above (Cortical and Neuronal targets) implies that bipolar CR+ interneurons do participate in disinhibitory but also feedforward and feed-back inhibitory circuits (Isaacson and Scanziani, 2011) of any cortical layer, column and area, especially when considering their high degree of colocalization with VIP (Porter et al., 1998; Reyes et al., 1998; Gonchar and Burkhalter, 2003; Caputi et al., 2009; Lee et al., 2013; Pfeffer et al., 2013; Pi et al., 2013). Whether the content of CR in these circuits is meaningful on its own, like it has recently been suggested for another calcium-binding protein (i.e., parvalbumin in the hippocampus; Donato et al., 2013), is currently unknown (but see Schwaller, 2014 for a recent review). The best understood function is now the disinhibitory action that is dependent on a unique circuit motif, i.e., targeting of other inhibitory interneurons, mostly SOM- (or CB-)expressing Martinotti cells. In the somatosensory system of mice, Lee et al. (2013) found that a specific projection from the whisker motor cortex excites VIP+ neurons and subsequently inhibits SOM+ neurons in the primary somatosensory cortex. This leads to a disinhibition of the apical dendrites of the pyramidal cells during exploratory behavior (Figure 6), probably acting as a “gate-opener” for the paralemniscal pathway (Gentet et al., 2012). A similar gate-opening mechanism might be functional in the primary auditory cortex, when animals learn to associate a tone stimulus with an aversive context (Pi et al., 2013).

#### Peptidergic neuromodulation

The expression of numerous neuropeptides in CR+ bipolar interneurons (Kubota et al., 1994, 2011; Kaneko et al., 1998; Taki et al., 2000), whose release requires a high level of neuronal activity (Zupanc, 1996; Baraban and Tallent, 2004), could also allow an activity-dependent fine tuning of the cortical network. For instance in the cat visual cortex, exogenously applied VIP had little or no effect on recorded neurons in the absence of visual stimulation, but enhanced their visual responses (Murphy et al., 1993; Fu et al., 2014). Consistently, by activating VPAC1 receptors and cAMP/PKA signaling, VIP reduced the slow AHP current and the tonic potassium current which regulates the excitability of hippocampal and cortical pyramidal neurons (Haug and Storm, 2000; Hu et al., 2011). Interestingly, CRF whose CRF1 receptors are also expressed by pyramidal neurons (Gallopin et al., 2006), induced an even more pronounced increase in cAMP/PKA signaling and modulation of potassium currents than VIP (Haug and Storm, 2000; Hu et al., 2011). Presumably because of the rapid desensitization of CRF1 receptors (Hauger et al., 2000), the action of CRF rapidly declined.

CR+ bipolar interneurons are also likely to enhance glutamatergic activity via NKB signaling (Figure 6). Indeed, NK-3 receptor, the most selective receptor for NKB (Shigemoto et al., 1990), is expressed by layer V pyramidal cells (Ding et al., 1996; Shughrue et al., 1996; Gallopin et al., 2006) and its activation depolarizes them (Stacey et al., 2002; Rekling, 2004; Gallopin et al., 2006).

In summary, to be operational, this complex peptidergic neuromodulation of the cortical circuit (Figure 6) requires a selective enhancement of CR+ bipolar neurons activity to achieve a substantial release of neuropeptides (Zupanc, 1996; Baraban and Tallent, 2004). This prerequisite could be met during specific brain states such as locomotion (Fu et al., 2014), which associates an increased activity of basal forebrain cholinergic neurons (Lee et al., 2005) and serotonergic neurons of the raphe (Wu et al., 2004) leading to the concomitant activation of excitatory receptors on CR+ bipolar neurons (see Subcortical inputs).

### Glio-vascular network

#### Non-neuronal targets

Only very few studies have investigated the association of CR+ interneurons with non-neuronal elements (Consonni et al., 2009). However, perivascular appositions of intrinsic cortical VIP+ and ChAT+ neurons, two markers frequently coexpressed in CR+ bipolar interneurons (Figure 4), are well documented in the rat cerebral cortex (Eckenstein and Baughman, 1984; Galea et al., 1991; Chédotal et al., 1994a,b; Paspalas and Papadopoulos, 1998; Fahrenkrug et al., 2000; Cauli et al., 2004). Different vascular compartments including pial and diving arterioles as well as capillaries are targeted by these terminals (Figure 6), which can be of conventional axonal or more unconventional dendritic origins. The precise examination of these perivascular appositions at the ultrastructural level revealed that VIP+ and ChAT+ varicosities are enriched in the vicinity of the vascular wall but systematically being separated from it by a thin astrocytic leaflet (Figure 6)(Chédotal et al., 1994a,b; Paspalas and Papadopoulos, 1998). These anatomical observations suggest that CR+ bipolar interneurons through glial and vascular interactions are likely to play a role in the control of cortical energy supply (see below).

#### Neurovascular coupling

VIP is a potent vasodilatory neuropeptide of pial and diving cortical arterioles (Wei et al., 1980; Itakura et al., 1987; Yaksh et al., 1987; Cauli et al., 2004) and the expression of VPAC1 receptors has been described in blood vessels (Martin et al., 1992; Fahrenkrug et al., 2000; Cauli et al., 2004). Furthermore, acetylcholine, but also GABA, induce vasodilatation of cerebral arterioles (Lee et al., 1984; Fergus and Lee, 1997). Numerous studies have reported an intimate association between VIP+/ChAT+ terminals of bipolar interneurons with cortical blood vessels (Eckenstein and Baughman, 1984; Galea et al., 1991; Chédotal et al., 1994a,b; Paspalas and Papadopoulos, 1998; Fahrenkrug et al., 2000; Cauli et al., 2004) (see Non-neuronal targets). Based on these observations, as early as in the 1980s and until recently, VIP+ bipolar neurons have been proposed to be involved in the control of regional cerebral blood flow (Eckenstein and Baughman, 1984; Magistretti, 1990; Buzsaki et al., 2007; Cauli and Hamel, 2010). Although, single cell stimulation of VIP+ bipolar neurons was sufficient to elicit vasodilatation of nearby diving arterioles, the messengers recruited in these vascular responses were undetermined (Cauli et al., 2004). Indeed, the contribution of endogenous VIP to the control of regional cerebral blood flow has been largely restricted by the lack of selective VIP receptors antagonists (Yaksh et al., 1987). The role of VIP in neurovascular coupling was recently questioned in studies showing that vascular responses induced by sensory or pharmacological stimuli efficiently activating VIP+/ChAT+ bipolar neurons were insensitive to VIP receptors blockade (Lecrux et al., 2011; Perrenoud et al., 2012b). It also is unlikely that acetylcholine released by VIP+/ChAT+ bipolar interneurons significantly contributes to the neurovascular coupling induced by sensory stimulation as these responses were insensitive to the blockade of muscarinic receptors (Lecrux et al., 2011) known to be involved in the vasodilatations induced by acetylcholine (Elhusseiny and Hamel, 2000; Kocharyan et al., 2008). GABA is another possible vasoactive messenger produced by bipolar interneurons since the activation of GABA-A receptors can indeed elicit vasodilation (Fergus and Lee, 1997) and their blockade impairs the neurovascular response to sensory stimulation (Lecrux et al., 2011). However, it is difficult to discriminate between a pure vascular effect of GABA from an alteration of the cortical network activity (Figure 6).

CRF is another putative candidate peptide for neurovascular regulation through its release from CR+ bipolar cells (Cauli and Hamel, 2010) (Figure 6). It is a vasodilator of blood vessels (De Michele et al., 2005) and its CRF1 receptors are expressed in blood vessel (Chalmers et al., 1995). To our knowledge, the contribution of CRF in neurovascular coupling has not been investigated and it may represent one of the yet undetermined vasodilatory messengers in the neurovascular response (Leithner et al., 2010; Liu et al., 2012). Interestingly, CRF is also expressed in a subpopulation of SOM+ interneurons (Gallopin et al., 2006; Kubota et al., 2011) but both SOM (Long et al., 1992) and the direct stimulation of SOM+ interneurons were shown to induce vasoconstrictions (Cauli et al., 2004). Given that SOM+ neurons are a major neuronal target of CR+ bipolar interneurons (see chapter 6.1.1), it is likely that CR+/VIP+ bipolar interneurons and CR+/SOM+ interneurons (Figure 6), via vascular and/or synaptic interactions, play opposite roles in the control of regional cerebral blood flow (Kleinfeld et al., 2011).

#### Neurometabolic coupling and gliotransmision

The axonal and dendritic terminals of VIP+/ChAT+ bipolar neurons, enriched in the vicinity of blood vessels, are physically separated from the vascular wall by a thin astrocytic leaflet (Figure 6), as already mentioned above (Chédotal et al., 1994a,b). Since astrocytes express VIP receptors (Martin et al., 1992), this suggests that bipolar interneurons may also activate astrocytic functions.

It is well established that astrocytes play a key role in cerebral metabolism, notably by storing blood glucose into glycogen (Cataldo and Broadwell, 1986; Magistretti, 1990; Allaman et al., 2011) but also by stimulating glycolysis and lactate release during neuronal activity (Pellerin and Magistretti, 1994; Ruminot et al., 2011; Choi et al., 2012), with lactate being a major oxidative energy substrate over glucose for neurons (Bouzier-Sore et al., 2003). Besides its role in energy metabolism astrocyte glucose is also the precursor of several gliotransmitters such as D-serine (Ehmsen et al., 2013) and possibly lactate (Barros, 2013) which play a key role in synaptic plasticity and memory formation (Panatier et al., 2006; Suzuki et al., 2011).

Interestingly, VIP, but not GABA, cholinergic agonists, somatostatin, CRF, or enkephalins, were shown to stimulate glycogenolysis in cortical slices (Magistretti et al., 1981, 1984; Magistretti, 1990). This indicates that VIP+/ChAT+ bipolar interneurons can specifically enhance the recruitment of astrocytic glycogen stores (Figure 6) via VIP receptors (Martin et al., 1992) and cAMP/PKA signaling (Magistretti and Schorderet, 1984). In addition to this short term effect occurring within minutes, VIP also transcriptionally promotes glycogen re-synthesis within hours (Sorg and Magistretti, 1992). By tightly regulating glycogen content, and therefore astrocytes, glucose metabolism VIP+ bipolar neurons might play essential roles in the regulation of energy metabolism but also memory (Buzsaki et al., 2007; Pi et al., 2013).

## Conclusions

More than 25 years ago, bipolar cells were considered to be enigmatic due to their rare occurrence, unusual synaptology, and unknown functions (Peters and Harriman, 1988). A big step forward in specifically studying the properties and functions of cortical inhibitory interneurons was the generation of specific mouse lines, first BAC-transgenics (Caputi et al., 2009), later Cre-driver lines (Taniguchi et al., 2011). The latter can be used now together with other exciting new methods, to precisely determine their inputs (via cre-dependent Rabies virus tracing; Choi et al., 2010; Fu et al., 2014) or outputs (via Cre-dependent channelrhodopsin-expressing vectors; Yizhar et al., 2011), both, *in vivo* and *in vitro*.

Eagerly awaiting these studies that will provide direct evidence for the functions of CR+ bipolar interneurons, summarizing the available -mostly indirect evidence coming from studies on VIP Cre interneurons that are likely to co-express CR- one can state that probably the bipolar neurons are amongst the most complex cells in the cerebral cortex because they seem to be involved in every major mechanism that has to be active to support successful neuronal computation. At a supportive level, they play a core role in controlling blood flow and energy metabolism, using a multitude of effector molecules and associated receptors. At a neuronal level, it seems that they are able to integrate local and distant cortical excitatory inputs, together with subcortical sensory and modulatory inputs from all major neurochemical systems that regulate functions like arousal, attention, reward, and sleep-wake cycles, to name but a few. All these inputs are integrated and conveyed as a processed output to the still badly characterized target neurons. However, assuming that CR+ interneurons share most of their properties with VIP+ interneurons, the now well-established “disinhibitory circuit motif” might be the core function of this neuron class. This function was so tightly connected to several behavioral correlates that the notion came up that inhibitory interneurons are not only “modulators” of the information processing by pyramidal cell circuits but do compute information themselves (Hangya et al., 2014; Karnani et al., 2014; Munoz and Rudy, 2014).

With all this new information in mind, we think it is safe to conclude that cortical inhibitory bipolar interneurons are not that enigmatic anymore as they appeared roughly 25 years ago.

### Conflict of interest statement

The authors declare that the research was conducted in the absence of any commercial or financial relationships that could be construed as a potential conflict of interest.
